# Impact of implementing a non-restrictive antibiotic stewardship program in an emergency department: a four-year quasi-experimental prospective study

**DOI:** 10.1038/s41598-020-65222-7

**Published:** 2020-05-18

**Authors:** Alessia Savoldi, Federico Foschi, Florian Kreth, Beryl Primrose Gladstone, Elena Carrara, Simone Eisenbeis, Michael Buhl, Giuseppe Marasca, Chiara Bovo, Nisar Peter Malek, Evelina Tacconelli

**Affiliations:** 1 Division of Infectious Diseases, Department of Internal Medicine I, German Center for Infection Research, University of Tübingen, Otfried Müller Straße 12, 72074 Tübingen, Germany; 2 Division of Infectious Diseases, Department of Diagnostic and Public Health, G. B Rossi University Hospital, University of Verona, P.le L.A Scuro 10, 37100 Verona, Italy; 3 Medical Department, Division of Nephrology and Internal Intensive Care Medicine, Charitè, Augustenburgerplatz 1, 13353 Berlin, Germany; 4 Division of Emergency Medicine, Department of Internal Medicine I, University of Tübingen, Otfried Müller Straße 12, 72074 Tübingen, Germany; 5 Institut for Microbiology, Virology and Hygiene, University Hospital Rostock, Schillingallee 70, 18057 Rostock, Germany; 6 Department of Infectious-Tropical Diseases and Microbiology, IRCCS Sacro Cuore Don Calabria Hospital, 37024 Negrar, Verona Italy; 7 Medical Direction, G. B Rossi University Hospital, University of Verona, P.le L.A Scuro 10, 37100 Verona, Italy; 8 Department of Gastroenterology, Hepatology and Infectious Diseases, Tübingen University Hospital, Tübingen, Germany

**Keywords:** Bacterial infection, Health policy

## Abstract

Antibiotic resistance is increasing worldwide. The implementation of antibiotic stewardship programmes (ASPs) is of utmost importance to optimize antibiotic use in order to prevent resistance development without harming patients. The emergency department (ED), cornerstone between hospital and community, represents a crucial setting for addressing ASP implementation; however, evidence data on ASP in ED are poor. In this study, a 4-year, non-restrictive, multi-faceted ASP was implemented in a general ED with the aim to evaluate its impact on antibiotic use and costs. Secondly, the study focused on assessing the impact on length of hospital stay (LOS), *Clostridioides difficile* infection (CDI) incidence rate, and mortality in the patients’ group admitted from ED to medical wards. The ASP implementation was associated with a reduction of antibiotic use and costs. A mild but sustained LOS decrease in all medical wards and a significant downward trend of CDI incidence rate were observed, while mortality did not significantly change. In conclusion, the implementation of our ED-based ASP has demonstrated to be feasible and safe and might clinically benefit the hospital admitted patients’ group. Further research is needed to identify the most suitable ASP design for ED and the key outcome measures to reliably assess its effectiveness.

## Introduction

The inappropriate prescription of antibiotics represents one of the most important preventable factors contributing to the spread of antibiotic resistance^1^. In order to address this issue, antibiotic stewardship programs (ASPs) have been developed with the aim of optimizing clinical outcomes while minimizing unintended consequences^2^ and have been successfully implemented in medical, surgical and intensive care units. Systematic reviews have shown, with convincing level of evidence, that the introduction of hospital wide ASPs results in reduction of antibiotic usage^3^, antibiotic resistance^4^, and adverse drug events, such as nephrotoxicity and Clostridioides difficile infections (CDIs)^4,5^.

There is a paucity of literature pertaining to ASPs in the emergency department (ED)^6–8^, likely because many of the strategies commonly adopted by ASPs may be difficult to be implemented in the ED^9^. There are logistical system- and provider-level issues making the ED a critical environment for addressing interventions to reduce the inappropriate antibiotic prescription rate^10^,including ED overcrowding^8^, high turnover of patients^9^, diminished continuity of care due to high variability of practitioners^11^, and therapeutic decisions made frequently by a single ED practitioner in a rapid decision making process often without meaningful microbiologic information^12^. Furthermore, since ED sits at the interface between community and hospital, the antibiotic selection in ED has the potential to affect the antibiotic usage of both the discharged and the admitted patients with important downstream implications. In fact, antibiotic regimens started in the ED are often maintained even when another clinician has assumed care of the patient^11^.

In 2013 a “call to action” was published by May *et al*., advocating the implementation of ASPs within the ED due to the far-reaching consequences that prescriptions in this setting might have on patient outcomes. The statement underlined the need to shift attention to EDs to define which of the multiple antibiotic stewardship strategies are most feasible in this setting^7^. In this context, the Centers for Disease Control and prevention promoted the development of the MITIGATE toolkit (A Multifaceted Intervention to Improve Prescribing for Acute Respiratory Infection for Adults and Children in Emergency Department and Urgent Care Settings), a six-core component framework for implementing non-restrictive intervention (mostly education and audit and feedback) in the ED^13^. Besides this example, very few guidance documents are available on this topic. Therefore, in response to this lack of evidence and in a bid to underline the relevance of the topic, the European Society of Clinical Microbiology and Infectious Diseases has recently started the process for developing clinical guidelines on ASPs in the ED.

Aim of this study was to assess the impact of a non-restrictive ASP intervention in a non-surgical ED of a tertiary university hospital on reducing the use of antibiotics and related costs. The secondary aim was to evaluate the clinical impact of the ED-based ASP intervention on the hospital-admitted patients’ group.

## Methods

### Study design and setting

We conducted a prospective quasi-experimental study using an interrupted time-series (ITS) analysis between 2014 and 2017. The study setting was the non-surgical ED of the University Hospital of Tübingen, a 1.513-bed tertiary care teaching hospital. The hospital includes different medical and surgical specialties: liver, kidney and bone marrow transplant units, a paediatric unit, a maternity ward, and a dialysis unit. The ED has 22 beds, equally distributed between the emergency room and the short term observation ward. The ED service is carried out by three personnel shifts. Two internal medicine physicians are permanently employed in the ED, whilst four senior internal medicine residents are on 6-12-month rotation. Three and two practitioners carried out the day and night shift, respectively. During the study period, there were yearly about 10.000 patient contacts, with approximatively 6000 hospitalizations. Before the ASP no official internal guidelines on antibiotic therapy and no routine infectious disease specialist visits were available for the ED.

### ASP implementation

The ASP was designed and carried out by a dedicated team, including two infectious diseases physicians, one infectious diseases resident, one clinical microbiology resident and two study nurses. The intervention model was exclusively non-restrictive. The ASP was composed of a one-year pre-intervention phase (Phase I), a 2-year multifaceted intervention phases (Phase II, 2015 and Phase III, 2016), followed by one year of post- intervention phase (Phase IV, 2017).

The ASP program included the following core elements:

*Prospective epidemiological and clinical data collection* (Phase I, 2014)

*Development and dissemination of guidelines on appropriate antibiotic empiric treatment* (Phase II, Jan-Aug 2015). The whole ASP team developed the internal guidelines based on hospital epidemiological resistance data, on clinical and epidemiological patients’ data collected in the Phase I, and on the most relevant national/international therapeutic guidelines regarding the main infectious diseases syndromes. The guidelines were made available in written pocket-sized format and distributed to the whole ED staff.

*Education* (Phase II, May-Dec 2015). The ASP team had weekly meetings with ED staff to discuss the validation of the guidelines and the local ecology. The ED staff carried out the on-line course “Antimicrobial Stewardship: Managing Antibiotic Resistance” promoted by the University of Dundee, United Kingdom and the British Society for Antimicrobial Chemotherapy (https://www.futurelearn.com/courses/antimicrobial-stewardship).

*Prospective audit and feedback* (Phase III, Jan- Dec 2016). At least two members of the ASP-team reviewed daily each antibiotic prescription in ED, in accordance with the guidelines. The following patient data were collected and entered into a database: age, gender, underlying comorbidities, diagnosis and antibiotic prescription(s), including name, posology, length and administration route, length of hospital stay (LOS) and mortality. Microbiological data on antibiotic resistance were also gathered. The feedback was provided through weekly newsletters, reporting the compliance rate with the guidelines, the antibiotic use (expressed in daily defined dose, DDD) and the microbiological isolates with the antibiotic resistance profile. The newsletters also included the most relevant publications of the month. Additionally, monthly meetings were held focusing on the discussion of the most relevant clinical cases in which antibiotics were deemed to be inappropriate. The participation of the ED team to these meetings was strongly supported. An example of the newsletter is displayed in the Supplementary Figure 1.

*Active infection diseases consultation service* (Phase III, Jan – Dec 2016): in the second phase a specialist consultation service conducted by three infectious diseases physicians were active 24 h seven days a week.

*Random audit and periodical feedback* (Phase IV, Jan- Dec 2017): a feedback was provided quarterly through newsletters briefly summarizing the antibiotic use.

The design of the ASP program and time line of the applied interventions are detailed in the Supplementary Figure 2.

### Outcomes measures and definitions

The primary outcome was the total monthly (oral and parenteral) antibiotic use in ED, measured as DDD per 100 patient days (DDD per 100PDs) according to the 2014 Anatomical Therapeutic Chemical (ATC) Defined Daily Dose Index delivered by the World Health Organization (https://www.whocc.no/atc_ddd_index/). Antibiotics were classified following the ATC therapeutic subgroup J01 (antibacterial for systemic use). The DDD data were retrieved from the hospital pharmacy records for the time-frame January 2014 - December 2017 and reflected the monthly amount of antibiotics dispensed to the ED. To capture potential shift in use, overall DDDs were also stratified into three groups (*Access, Watch* and *Reserve*) in accordance with the 2019 World Health Organization (WHO) AWARE classification^14^. Secondary outcomes were: yearly antibiotic costs calculated in EUROs/100 PDs, and clinical outcomes including LOS, monthly CDI incidence (calculated as number of events per 100 PDs) and in-hospital all-cause mortality. The clinical outcomes were measured in the inpatients group, that includes the patients enrolled in ED ASP and admitted to the hospital. These patients were followed until hospital discharge or death.

### Statistical analysis

Statistical analyses were conducted using an ITS model in accordance with the Cochrane Effective Practice and Organization Care recommendations (EPOC)^15^. The ITS included four period segments: pre-intervention phase I (January to December 2014), intervention phase II (January-December 2015), intervention phase III (January-December 2016) and post-intervention phase IV (January- December 2017). Estimates for regression coefficients corresponding to the effect sizes of a change in level and a change in trend along the study phases were obtained. A change in level was defined as the difference between the observed level immediately post-ASP and the predicted level by the pre-ASP trend. A change in trend was defined as the difference between the pre and post-ASP slopes. Newey-West standard errors with a maximum lag of 2 was considered for the autocorrelation structure. The other outcomes were analysed using Chi-square test and ANOVA. The trend of antibiotic costs was studied using Poisson regression. A p value less than 0.05 was regarded as significant. All statistical analyses were carried out using STATA version 14.2 (Stata Corp LLC, Texas). The methods were carried out in accordance with relevant guidelines and regulations. The informed consent was taken from all ED health-care providers involved in the study. The Ethics Committee of the University of Tübingen declared that no ethics approval was necessary since the study was considered as a quality improvement intervention.

## Results

Overall, the ASP intervention included 42886 patients evaluated in ED receiving an antibiotic treatment during the whole study period. The yearly admission rate to the hospital was similar in the four phases (Table 1).

**Table 1:** Number of patients received antibiotic treatment in ED and admission rate per year.

Study phase	Phase I (2014)	Phase II (2015)	Phase III (2016)	Phase IV (2017)	Total
Patient receiving antibiotics in ED, n	10647	10565	10840	10834	**42886**
Discharged patients, n (%)	3871 (37%)	4317 (41%)	4009 (37%)	3911 (37%)	**16108 (37.5%)**
Hospital admitted patients*, n (%)	6776 (63%)	6322 (59%)	6831 (63%)	6923 (63%)	**26852 (62.5%)**

ED: emergency department.

*Patients were admitted to one of the following wards: (a) gastroenterology, hepatology and infectious diseases; (2) hematology, pneumology and oncology; (3) cardiology; (4) nephrology, endocrinology and angiology; and (5) intensive care unit.

**Figure 1:**
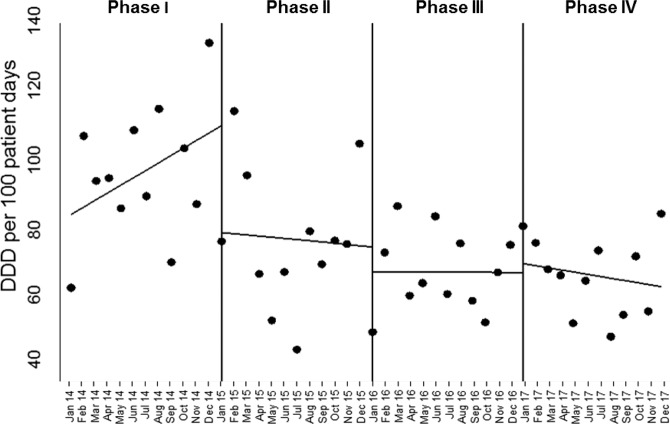
Effect of the antibiotic stewardship implementation on the overall antibiotic use in DDD per 100 patient days in the study periods. The solid line represents the estimated slope by the segmented regression model. Abbreviation: DDD: daily defined dosis.

As for the overall antibiotic use, the ITS analysis showed a non-significant decrease of 31.12 DDD/100 PDs (confidence interval (CI) 95% −67.50 to 5.27, p 0.092) at the beginning of phase II and a further decrease of 7.20 DDD/100 PDs (CI 95% −40.94 to 26.54, p 0.669) at the beginning of phase III (Table 2, Fig. 1). When categorizing the antibiotic use in accordance with the 2019 WHO AWARE classification^14^, an homogeneous DDD reduction in each of the AWARE antibiotic group was observed (Supplementary Table 1).

**Table 2:** Results of the interrupted time series analysis comparing the overall antibiotic use and the incidence rate of C. difficile infection in the four study phases.

OVERALL ANTIBIOTIC USE IN DDD per 100 patient days						
**Study phase**	Baseline level (β0)	Baseline slope	Change in level (CI 95%)	P value	Change in slope (CI 95%)	P value
Phase I	83.68	2.16	—	—	—	—
Phase II			−31.12 (−67.50 to 5.27)	0.092	−0.35 (−4.31 to 3.62)	0.861
Phase III			−7.20 (−40.94 to 26.54)	0.669	−0.02 (−2.16 to 2.11)	0.983
Phase IV			2.60 (−14.40 to 19.60)	0.759	−0.61 (−2.71 to 1.49)	0.562
**INCIDENCE RATE OF** ***C. DIFFICILE*** **INFECTIONS per 100 patient days**						
**Study phase**	Baseline level (β0)	Baseline slope	Change in level (CI 95%)	P value	Change in slope (CI 95%)	P value
Phase I	0.80	0.12	—	—	—	—
Phase II	—	—	−0.45 (−0.52 to 0.43)	0.847	0.04 (−0.01 to 0.09)	0.133
Phase III	—	—	−0.23 (−0.75 to −0.29)	0.381	−0.06 (−0.10 to – 0.01)	**0.014**
Phase IV	—	—	0.11 (−0.31 to 0.54)	0.588	0.002 (−0.05 to 0.05)	0.946

DDD: daily defined doses. CI: confidence interval.

A significant decrease in the mean yearly antibiotic cost per 100 PDs was recorded throughout the study phases, from 691.5 EUROs/100 PDs (standard deviation, SD: 263 EUROs/100 PDs) in the phase I, to 358.7 EUROs/100 PDs (SD: 189 EUROs/100 PDs) in the phase II, 262.5 EUROs/100 PDs (SD: 162 EUROs/100 PDs) in phase III and 263.3 EUROs/100 PDs (SD: 162 EUROs/100 PDs) in the phase IV (p < 0.001).

Overall, 26852 patients out of 42886 (62.5%) were admitted to the hospital wards (Table 1).

The ASP implementation has determined an overall decrease of LOS of the inpatients group admitted in all the five medical wards throughout the study phases. A statistical significant LOS decrease by 0.5 days, 0.7 days and 0.6 days was recorded in the gastroenterology, hepatology and infectious diseases ward (LOS phase I: 4.6 days, SD 5.1 days; LOS phase IV: 4.1 days, SD 5.1 days; p < 0.0001), in the hematology, pneumology and oncology ward (LOS phase I: 8.4 days, SD 7.3 days; LOS phase IV: 7.7 days, SD 7.3 days, p < 0.0001), and in the nephrology, endocrinology and angiology ward (LOS phase I: 6.3 days, SD 6.8 days; LOS phase IV: 5.7 days, SD 6.8 days; p 0.006) respectively (Table 3).

**Table 3:** Comparison of mean monthly antibiotic costs and patient related outcomes in the ED department in the four study phases.

Variable	Phase I (2014)	Phase II (2015)	Phase III (2016)	Phase IV (2017)	p value*
Mean antibiotic costs euro/100 patient days (SD)	691.5 (263)	358.7 (189)	262.5 (162)	263.3 (162)	**0.001**
DDD (SD)	109.39 (20.27)	112.3 (34.69)	91.23 (18.69)	94.7 (20.00)	
In hospital all-cause mortality (n, %)	213 (3.3)	238 (3.7)	259 (2.4)	224 (2.1)	0.094
Mean length of hospital stay, days (SD) by ward					
Gastroenterology, hepatology and infectious diseases	4.6 (5.1)	4.6 (5.1)	4.2 (5.1)	4.1 (5.1)	**<0.0001**
Hematology, pneumology and oncology	8.4 (7.3)	8.2 (7.3)	7.6 (7.3)	7.7 (7.3)	**<0.0001**
Cardiology	4.5 (5.9)	4.5 (5.9)	4.7 (5.9)	4.4 (5.9)	0.112
Nephrology, endocrinology and angiology	6.3 (6.8)	6.2 (6.8)	5.8 (6.8)	5.7 (6.8)	**0.006**
Intensive care unit	2.2 (14.0)	2.0 (14.0)	2.2 (14.0)	2.0 (14.0)	0.948

DDD: defined daily doses, SD: standard deviation.

*p value for antibiotic costs computed using Poisson regression; p value for mortality computed using Chi-square test; p value for LOS computed using ANOVA.

The ITS analysis of CDI incidence showed a decreasing trend in both the intervention phases. From the phase II (change in level −0.45 CI 95% −0.52 to 0.43, p 0.847), the reduction of the incidence was more remarkable during the phase II, in which the change in level further decreased by 0.23% (CI 95% −0.75 to 0.29, p 0.381) and the change in trend achieved the statistical significance (change in slope −0.06, CI 95% −0.10 to −0.01, p 0.014) (Table 2).

The implementation of the ASP program was not accompanied by significant negative effects in terms of mortality. During the study period, the all-cause mortality rate remained stable: 3.3% (213/6776) in phase I, 3.7% (238/6322) in phase II, 2.4% (259/6831) in phase III and 2.1% (224/6923) in phase IV (Table 3).

## Discussion

Our study demonstrated that a 4-year non-restrictive multifaceted ASP program applied in ED setting may reduce the overall antibiotic use without adversely affecting mortality. The ASP implementation was associated with a significant decrease of the antibiotic costs by two thirds after the intervention; starting from 691.5 euro per 100 patient days in the pre-intervention phase, the cost has decreased quickly by two thirds in the following phases. Among the inpatient group, a significant reduction of both LOS and CDI incidence rate has been shown, pointing out that the intervention applied in ED might have an impact downstream on other medical areas of the hospital.

Notwithstanding the several challenges hampering a successful ASP implementation, our ED-based ASP intervention has been shown to be feasible and efficacious even when no restrictive measures were adopted. The use of an entirely persuasive approach represents, in fact, a relevant strength of our ASP. In accordance with a Cochrane systematic review, the non-restrictive strategy, embedding a high potential for educational opportunities, usually results in more sustained positive effects on the clinicians’ professional practice, in comparison with the restrictive approach^16,17^. In our study, the constant decrease of the overall antibiotic use, observed in each of the AWARE group (*Access, Watch* and *Reserve*), alongside the antibiotic costs might reflect a cumulative effect of the various persuasive interventions implemented stepwise. Worthy of note, the greatest decline of both outcomes was observed in phase III. The intensified “prospective audit and feedback” approach, the core element of this phase, may have played a crucial role in enhancing the clinicians’ knowledge and adherence to the guidelines and consequently to an improved appropriateness of prescription^18,19^.

The positive impact of ASPs on reducing the antibiotic use has been widely assessed in several inpatient settings^20–22^. However, very little literature is available for the ED. A German study observed a statistically significant decrease of the overall mean antibiotic use by 22% after the implementation of a non-restrictive 6-year ASP in a large academic ED^7^. A similar finding was reported in a general surgery ED in Italy. The implementation of a stewardship bundle based on education and diffusion of hospital guidelines for surgical prophylaxis and infections led to a significant drop of the antibiotic use by 18%^23^.

These findings, although promising, should be interpreted and compared with caution. The main issue, not hindering a reliable comparison of antibiotic use within and across hospitals, resides in the lack of standardized quantity metrics for antibiotic use, which largely differ across similar settings and providers, and for specific medical populations^24^. The most common adopted metric, the DDD, is regarded as highly inaccurate^25^ and might be discordant for several frequently prescribed antibiotics, since the administered dosage in clinical practice differs from the DDD suggested by the WHO^25,26^. An addtional common metric, the days of therapy (DOT), also poses challenges in its application in the context of the ED. First, DOT requires patient-level data that might not be obtainable in all institutions. Second, DOT computation relies on calendar days during which the patient receives antibiotic(s) and, consequently, DOT is strongly influenced by patient’s time of admission.

In order to address the issue, innovative standardized evidence-based metrics for antibiotic use have been proposed^27,28^. Among these, the Standard Antimicrobial Administration Ratio (SAAR), developed by Centers for Disease Control and Prevention, seems to be the most feasible. By aggregating various patients care locations and antibiotic categories, the SAAR enables a risk-adjusted antibiotic use comparison across multiple hospitals. However, this metric is not easily applicable in European hospitals, due to the different structure of healthcare systems and the lack of shared databases with accessible electronic health records^28^.

The ASP implementation led to a significant drop in antibiotic cost by two third along the whole study period. Although not specifically assessed in the ED setting, inpatients ASPs have led to remarkable cost savings for health systems. A meta-analysis published in 2016 by Karanika *et al*., including five studies, described a cost reduction of 33.9% after ASP implementation^3^. Notably, the impact of ASPs implementation on costs saving could be even greater, despite not reliably measurable. In fact, beyond the costs referred to the direct costs of the antibiotic agents, there are several indirect expenditures that are supposed to drop alongside;^29^ such as from the adverse drug events^30^.

Since the ED represents the cornerstone between community and hospital setting, we hypothesized that an appropriate antibiotic selection in ED might have a relevant impact along the entire care continuum and ED patients who were admitted to hospital would intuitively benefit. The reduction of both LOS and CDI incidence rate observed in the medical wards seems in fact to support the concept of “downstream effects”.

With regard to the LOS, a mild but sustained decrease has been shown in all medical wards throughout the study phases. Although some confounders affect this measure, the improvement of the patient care in ED, result of our ASPs, might partly explain this finding. As observed in the clinical practice, the antibiotic therapies started in ED are frequently kept unaltered during the whole hospital stay of the patients, regardless the change of healthcare providers, mainly because of their reluctance to deescalate or even to discontinue^11^. From this perspective, the upstream selection of appropriate antibiotic treatment in ED might have a positive effect downstream in the hospital wards, leading to reduced number of antibiotic starts, shorter treatment lengths and earlier switch-to oral administration.

The CDI incidence rate showed a downward trend throughout the study phases. Interestingly, the protective effect of ASPs on CDI rate has been widely described for restrictive ASP interventions focusing on the “high risk antibiotic classes”^31–33^, whilst the efficacy of entirely non-restrictive ASP implementation has been much less investigated^34,35^.

It is hard to evaluate how likely the CDI reduction could be directly related to the ASP implementation, since it was implemented in a different setting and, more importantly, several other co-interventions (e.g. infection control policies, hand hygiene) have been regularly employed in the hospital during the study period.

Notably, the reduction of antibiotic use was greater in Phase II, whilst the greater reduction of CDI incidence was recorded in Phase III. Since the development of CDIs is strongly related to prolonged antibiotic use^36^, it could be hypothesized that the improvement of antibiotic management might preserve the gut microbiota^37^, resulting belatedly in a reduction of CDI.

A strength of our study was the use of a Cochrane validated method to measure the antibiotic use, providing the best evidence for evaluating ASP in a quasi-experimental research setting, when a randomized trial is not applicable^38^.

Nonetheless, this study had some limitations. First, the single-center nature of the study might limit its generalizability to other ED settings, especially with different local antibiotic resistance rates. Second, the relatively small amount of DDDs per antibiotic classes did not allow to perform a reliable subgroup analysis^39^. Third, the lack of a control group not receiving the intervention did not allow appraising confounders and counterfactual outcomes.

In conclusion, the implementation of our multi-faceted non-restrictive ED-based ASP has demonstrated to be feasible and safe and may clinically benefit the hospital admitted patient group acting on LOS and CDI incidence rate. More research is needed to define the most appropriate ASPs design for the ED and the most suitable key outcome measures to reliably assess its effectiveness.

## Supplementary information

Supplementary Information.
