# Supplementary material for: Impact of implementing a non-restrictive antibiotic stewardship program in an emergency department: a four-year quasi-experimental prospective study
Source: Sci Rep. 2020 May 18;10:8194. doi: 10.1038/s41598-020-65222-7 (PMC7235006; doi:10.1038/s41598-020-65222-7)
Supplement: Supplementary file 1 — Supplementary Information. [file 41598_2020_65222_MOESM1_ESM.docx]

**Supplementary material of** **“Impact of implementing a non-restrictive antibiotic stewardship program in an emergency department: a four-year quasi-experimental prospective study”.**

Alessia Savoldi, Federico Foschi, Florian Kreth, Beryl Primrose Gladstone, Elena Carrara, Simone Eisenbeis, Michael Buhl, Giuseppe Marasca, Chiara Bovo, Nisar Peter Malek, Evelina Tacconelli

**Supplementary Table 1: Comparison of yearly mean antibiotic DDD stratified by AWARE antibiotic group in the before-after analysis**

| **Antibiotic use expressed as DDDs/100 patient-days (standard deviation)** | | | | | |
| --- | --- | --- | --- | --- | --- |
| ***AWARE Antibiotic group*** | **Phase I** | **Phase II** | **Phase III** | **Phase IV** | **p value** |
| **ACCESS** | 64.87 (16.12) | 51.15 (18.48) | 33.04 (14.56) | 46.44 (12.34) | 0.18 |
| **WATCH** | 67.50 (13.23) | 79.17 (20.24) | 69.56 (12.99) | 56.10 (14.18) | 0.99 |
| **RESERVE** | 7.73 (1.24) | 0.27 (0.92) | 2.79 (3.21) | 3.40 (2.76) | **<0.001** |
| **Access group:** Cefazolin, Amoxicillin, Ampicillin, Amoxicillin/Clavulanate, Ampicillin/Sulbactam, Flucoxacillin, Cotrimoxazol, Sulfadiazin, Metronidazole, Doxycicline, Clindamycin.  **Watch group**: Cefepim, Ceftazidim, Ceftriaxon, Piperacillin/Tazobactam, Ertapenem, Meropenem, Ciprofloxacin, Levofloxacin, Moxifloxacin, Vancomycin, Azithromycin, Clarithromycin, Erithromycin.  **Reserve group**: Daptomycin, Fosfomycin, Linezolid, Tigecyclin.  The antibiotic use by class was computed using Poisson regression. | | | | | |

**Supplementary figure 1: Example of newsletter**


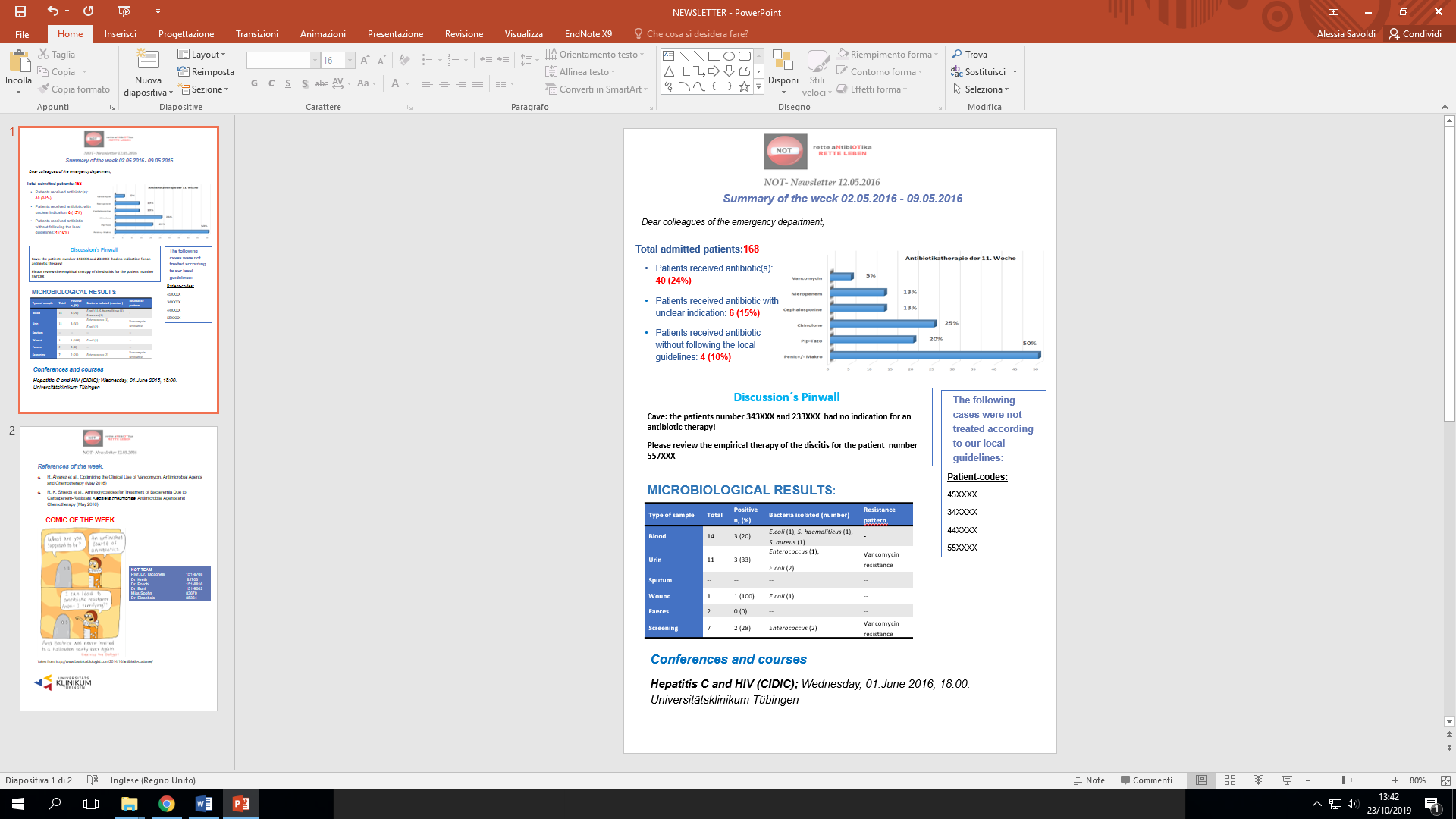


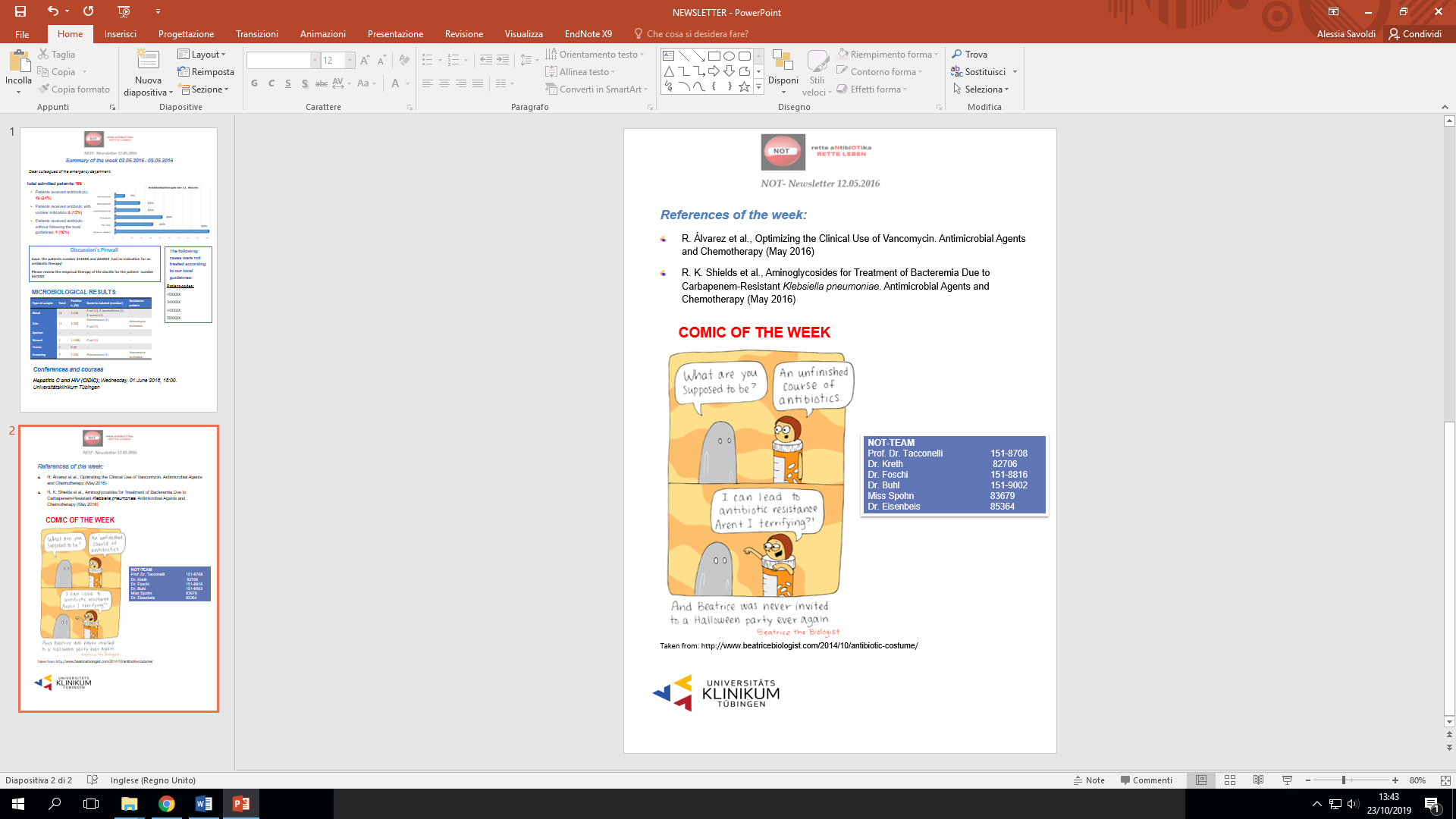


**Supplementary figure 2: Design of antibiotic stewardship program and timeline of the applied interventions**

**
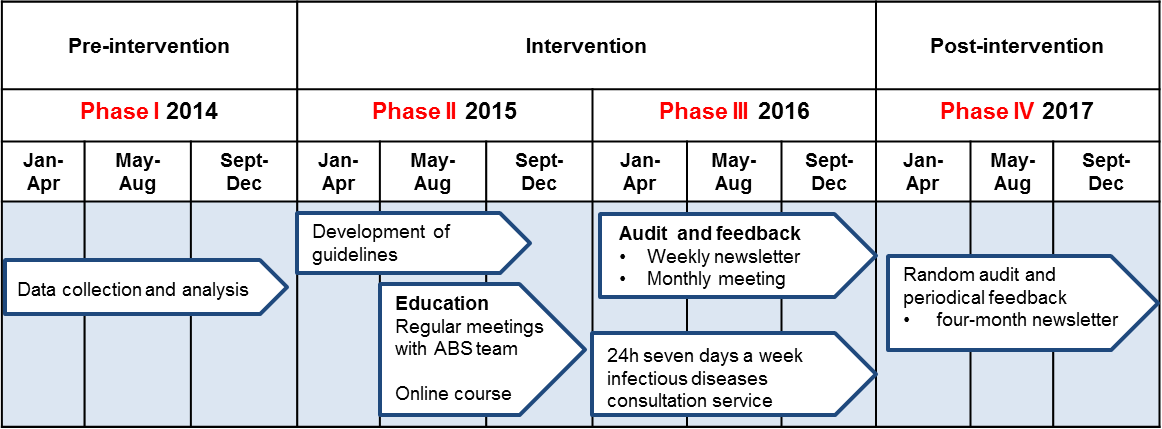
**
